# Improved Detection of Tuberculosis and Multidrug-Resistant Tuberculosis among Tibetan Refugees, India

**DOI:** 10.3201/eid2203.140732

**Published:** 2016-03

**Authors:** Kerry L. Dierberg, Kunchok Dorjee, Fulvio Salvo, Wendy A. Cronin, J’Belle Boddy, Daniela Cirillo, Tsetan Sadutshang, Richard E. Chaisson

**Affiliations:** Johns Hopkins University School of Medicine, Baltimore, Maryland, USA (K.L. Dierberg, J.’B. Boddy, R.E. Chaisson);; Tibetan Delek Hospital, Dharamsala, India (K. Dorjee, T. Sadutshang);; Central Tibetan Administration Department of Health, Dharamsala (K. Dorjee, T. Sadutshang);; San Raffaele Scientific Institute, Milan, Italy (F. Salvo, D. Cirillo);; Maryland Department of Health and Mental Hygiene, Baltimore (W.A. Cronin)

**Keywords:** tuberculosis and other mycobacteria, tuberculosis, TB, multidrug-resistant tuberculosis, bacteria, incidence, prevalence, active case finding, rapid molecular diagnostics, Xpert MTB/RIF, refugees, Tibet, India

## Abstract

The incidence of tuberculosis (TB) among Tibetan refugees in India is 431 cases/100,000 persons, compared with 181 cases/100,000 persons overall in India in 2010. More than half of TB cases in these refugees occur among students, monks, and nuns in congregate settings. We sought to increase TB case detection rates for this population through active case finding and rapid molecular diagnostics. We screened 27,714 persons for symptoms of TB and tested 3,830 symptomatic persons by using an algorithm incorporating chest radiography, sputum smear microscopy, culture, and a rapid diagnostic test; 96 (2.5%) cases of TB were detected (prevalence 346 cases/100,000 persons). Of these cases, 5% were multidrug-resistant TB. Use of the rapid diagnostic test and active case finding enabled rapid detection of undiagnosed TB cases in congregate living settings, which would not have otherwise been identified. The burden of TB in the Tibetan exile population in India is extremely high and requires urgent attention.

## Abstract 2

The prevalence of TB is extremely high in this population and requires urgent attention.

Tuberculosis (TB) continues to be one of the leading causes of death worldwide, and the largest prevalence of this disease in Asia (59%) and Africa (26%) (*1*). In 2014, a total of 23% of all incident TB cases were in India and 10% were in China. Approximately 4% of all new TB cases and 20% of previously treated cases are multidrug-resistant TB (MDR TB, characterized by resistance to isoniazid and rifampin), and >50% of these cases are in India, China, and Russia (*1*).

Refugee populations are known to be at increased risk for TB. This finding is believed to be caused, in part, by increased risks for malnutrition and overcrowding, which lead to increased susceptibility to and transmission of TB (*1*–*3*). Limited data for the incidence of TB among Tibetan refugees has demonstrated that it is among the highest in the world. In the mid-1990s, the incidence of TB in Tibetans living in India was estimated to be 835–1,700 cases/100,000 persons (*2*,*4*). Studies performed among Tibetan refugees in Minnesota, USA, and Toronto, Ontario, Canada, showed positive rates of 98% and 97%, respectively, for tuberculin skin tests, which indicated high rates of latent TB infection (*5*,*6*). In 2009, the incidence of TB in Tibetan refugees in New York, NY, USA was 561 cases/100,000 persons (New York City Department of Health and Mental Hygiene, unpub. data), which was 10-fold higher than the incidence for natives of India and China (*7*).

In 2010, the reported incidence rate of TB among Tibetans living in India was 431 cases/100,000 persons (Central Tibetan Administration Department of Health TB program [CTA DOH], unpub. data). More than half of cases were in students, monks, and nuns who live in congregate settings, where the potential exists for high rates of TB and MDR TB transmission. An estimated 90% of all new cases were in persons <35 years of age and ≈10% of these persons had MDR TB. The prevalence of HIV among TB patients was <1%. In 2011, the rate was similarly high (412 cases/100,000 persons; CTA DOH, unpub. data), much higher than the overall TB incidence of 181 cases/100,000 persons in India (*1*).

TB REACH is an initiative of the Stop TB Partnership, supported by the Canadian International Development Agency, that seeks to find innovative approaches for improving TB case detection in populations at high risk for this disease and with limited access to TB services (*8*). Historically, TB is diagnosed by passive case detection, which relies on symptomatic persons to seek care and the healthcare system to detect the disease. In settings where the incidence of TB is high and health services are weak, these limitations results in prolonged illness in patients and ongoing transmission of undetected TB.

Active case finding (ACF) for TB in such settings can identify persons with disease earlier than would occur under routine services and reduce illness and transmission (*9*). Given previously documented high TB incidence rates in the Tibetan refugee population and increased risk for TB in persons living in congregate living settings, we sought to improve case finding in Tibetans in India. We received support from TB REACH. Our main objective was to increase TB and MDR TB case detection rates through ACF in Tibetan congregate living centers in India, where the risk for TB is high.

## Methods

### Study Sites and Population

The study was conducted from September 2011 through March 2013 and approved by the Institutional Review Board of Johns Hopkins Medicine (Baltimore, MD, USA) and the Ethics Committee at Tibetan Delek Hospital (Dharamsala, India). We performed ACF for TB in Tibetan residential schools, monasteries, and nunneries in Himachal Pradesh, Karnataka, and Uttarakhand states in India, and in the Tibetan Reception Center in Dharamsala, a dormitory-style facility for newly arrived refugees from Tibet. In Karnataka, ACF was conducted among residents of congregate facilities and Tibetan households in Tibetan settlements in Bylakuppe and Mundgod. On the basis of a census conducted in 2009, the total population of Tibetans living in India is estimated to be 94,203 (*10*). The target population for this study, those living in congregate facilities, is estimated to be 53,150. However, more detailed population numbers for each congregate living setting were not available.

### Patient Screening and Enrollment

Before each ACF outreach activity, a member of the study team contacted the responsible administrator at every school, monastery, and nunnery to arrange dates and times for TB screening. Verbal consent was obtained from every participant before screening and enrollment. All children >12 years of age were screened at schools; children <12 years of age were screened if they had symptoms. The head nurse or teacher provided consent for screening. Each person was screened for symptoms of TB by using World Health organization (WHO) screening criteria (cough >2 weeks, fever, night sweats, or weight loss of any duration) through verbal or written questionnaires (*11*).

At the schools, a questionnaire with WHO symptom criteria was distributed to every student and staff member. At the smaller (<1,000 persons) monasteries and nunneries, each person was asked by study staff if they had 1 of these symptoms. Study staff interviewed any person who reported having >1 symptom. All persons previously given a diagnosis of TB and currently receiving treatment were excluded. At monasteries and nunneries that had >1,000 persons, an educational lecture was conducted for all residents. After being given information for signs and symptoms of TB, residents were asked to complete a questionnaire and notify project staff if they had >1 symptoms of TB. These persons were then interviewed.

The estimated total population screened was determined by using the most recent enrollment numbers at the Tibetan schools, monasteries, and nunneries. All newly arrived refugees at the reception center were screened and tested through interview by study staff.

All persons who were close contacts with someone known to have TB or MDR TB or who had been previously given treatment for TB were screened for symptoms of TB and received diagnostic testing (chest radiograph only versus chest radiograph and Xpert MTB/RIF test [Cepheid, Sunnyvale, CA, USA]). Contact information was obtained from school medical records, individual medical books (records), and patient interviews. A close contact was considered a person who lived in the same home, slept or studied in the same room, or spent time indoors with a person with TB.

Data were collected by use of a structured questionnaire for all persons with a history of TB, contact with a person with TB, MDR TB, or with symptoms of TB. Data collected included demographic information (age, sex, place of birth, date of arrival from Tibet), history of TB contact, smoking status, and history of TB. Persons with a history of TB were asked about the date of diagnosis, disease site, treatment duration, and treatment completion. TB contact history was further defined as recent (within the previous 6 months) or remote (>6 months) contact. Validation of data on previous TB was performed by personal medical records (individual medical information) carried by each person or TB treatment cards whenever possible. Symptoms were again reviewed and diagnostic testing was ordered as needed. When other members of the Tibetan community came to a site where ACF was being conducted, they were interviewed by using the same protocol described and enrolled in the database.

### Diagnostic Testing

Diagnostic testing included chest radiography or sputum testing with routine acid-fast bacilli (AFB) microscopy or Xpert MTB/RIF on the basis of a diagnostic algorithm developed for this study (Figure). The Xpert MTB/RIF is a rapid molecular diagnostic assay that can detect *Mycobacterium tuberculosis* in sputum and other specimens in <2 hours, and that identifies mutations in the *rpoB* gene associated with rifampin resistance. This cartridge-based system has high sensitivity (70%–90%) for *M. tuberculosis* and detects rifampin resistance with an accuracy >95% (*12*,*13*). As part of this study, an Xpert MTB/RIF apparatus was installed in the main health centers in the Bylakuppe and Mundgod Tibetan settlements. One Xpert MTB/RIF apparatus was already in place at Tibetan Delek Hospital.

**Figure:**
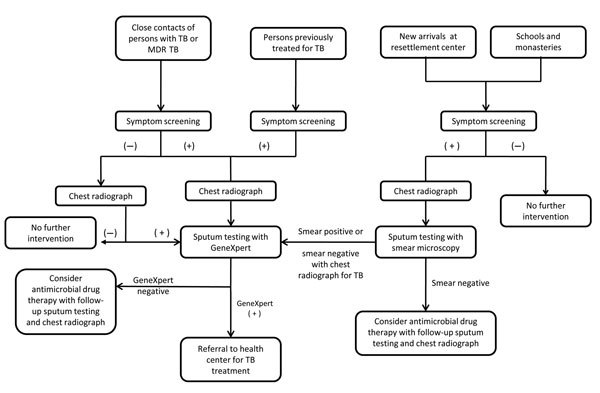
Diagnostic algorithm for improved detection of tuberculosis (TB) and multidrug-resistant TB (MDR TB) among Tibetan refugees, India. GeneXpert, Xpert MTB/RIF test (Cepheid, Sunnyvale, CA, USA). MTB, *Mycobacterium tuberculosis*; RIF, rifampin; –, negative; +, positive.

For all persons who were close contacts of a person with TB or MDR TB or had been previously given treatment for TB, the following diagnostic sequence was performed. First, screening was performed by using WHO symptom criteria and a written questionnaire. Second, persons who were symptomatic (>1 symptoms) received a chest radiograph and sputum testing (if able to produce sputum) by Xpert MIB/RIF. On the basis of these test results, the following actions were taken. If a person had a positive result for Xpert MTB/RIF, the person was referred to the nearest treatment center for TB treatment. If a person had a chest radiograph suggestive of TB and a negative or unavailable result for Xpert MTB/RIF, the person was referred to a physician for further evaluation. If a person had a negative results for Xpert MTB/RIF and chest radiograph, the person was referred to a physician for appropriate treatment or further diagnostic testing. Finally, all persons who were asymptomatic contacts of a TB patient received a chest radiograph. If active disease was suspected, further diagnostic testing was performed as described.

For persons who were not given previous TB treatment and who had no contact with a person known to have TB, the same symptom criteria were used; symptomatic persons received a chest radiograph and sputum testing with AFB smear microscopy, if they were able to produce sputum. All chest radiographs and sputum tests were performed at the nearest Tibetan health center. If the chest radiograph suggested TB but the AFB microscopy result was negative, testing by using Xpert MTB/RIF was then performed.

Persons who met symptom criteria or had chest radiograph findings suggestive for TB but who had negative results for AFB smear and Xpert MTB/RIF were given antimicrobial drugs for treatment of pneumonia. Patients with persistent symptoms were then retested by using chest radiography and sputum testing with Xpert MTB/RIF. If sputum results were negative but chest radiograph and symptoms still suggested TB, patients were identified as having TB and given anti-TB therapy.

Regardless of the diagnostic methods used, all persons given a diagnosis of TB were recorded in the study database. These patients were given their test results and referred to the nearest TB treatment center to initiate anti-TB treatment. The study database was cross-referenced against the records of the health facility to which the patient was referred. If referred patients were not found in the health facility records, then the patient was contacted to investigate the reasons why this omission occurred (e.g., failure of the patient to seek follow-up treatment, accessed a different health facility) and treatment was ensured for all patients given a new diagnosis.

### Data Collection and Statistical Analysis

Data for study forms and laboratory reports were entered into a Microsoft (Redmond, WA, USA) Excel database by project staff. Data analysis was performed by using descriptive summaries, χ^2^ or Fisher exact tests, and *t*-tests, as appropriate.

## Results

During September 2011–March 2013, a total of 27,714 persons were screened for symptoms of TB at 21 Tibetan schools, 36 monasteries/nunneries, and the reception center for newly arrived refugees. These activities were conducted in Himachal Pradesh, Karnataka, and Uttarakhand states. Because data on age and sex for Tibetan school populations were not available, it was not possible to determine the age and sex distributions of the total population screened in this study. However, most (64%) of the persons screened were male. Of the total population screened, 55.0% (15,291) were residents of monasteries, and 1.6% (437) were refugees screened at the reception center.

A total of 3,830 (13.8%) persons with symptoms of TB or who were asymptomatic contacts of someone with TB were further evaluated and received chest radiography, sputum testing by AFB microscopy, Xpert MTB/RIF testing, or some combination (Table 1). Of 3,830 persons evaluated, 2,464 (64%) were male and 1,366 (36%) were female. Median age was 18 years (range 3–86 years) (Table 1); a total of 52.6% (2,016) were <18 years of age and 89% (3,413) were <30 years of age.

**Table 1:** Characteristics of 3,830 Tibetan refugees in India screened for tuberculosis*

Characteristic	No. (%)
Sex	
M	2,464 (64.3)
F	1,366 (35.7)
Age, y	
<18	1,633 (42.6)
18–24	1,363 (35.6)
25–34	529 (13.8)
35–44	194 (5.1)
45–54	53 (1.4)
>55	58 (1.5)
Enrollment group	
Students	2,118 (55.3)
Monks/nuns	1,155 (30.2)
Reception center personnel	438 (11.4)
Other (community members, contacts)	73 (1.9)
Ethnicity	
Tibetan	3,585 (93.6)
Other (India, Nepal, Bhutan, Mongolia)	245 (6.4)
Risk factors	
History of TB	391 (10.2)
Any history of TB contact	2,221 (58.0)
Recent close TB contact (<6 mo)	1,883 (49.2)
No known TB contact	1,609 (42.0)

*TB, tuberculosis.

Most (93.6%) persons evaluated were Tibetan; there were smaller numbers of persons from India, Bhutan, Nepal, and Mongolia. Of the persons enrolled and tested, 47% were born in Tibet and had immigrated to India. Exposure to TB was common; 49.2% reporting having close contact with someone with TB or MDR TB in the previous 6 months, and 58.0% reporting having close contact with a TB patient at any time.

TB was diagnosed in 96 (2.5%) of 3,830 persons evaluated (346 cases/100,000 persons in the surveyed population). When community members not resident in schools, monasteries, and the reception center were excluded from this analysis, the prevalence of undiagnosed TB among persons tested was 2.3% (85/3,757) or 307 cases/100,000 persons in the screened population. Of the 96 cases, 77 (80.0%) were in male participants and 19 (20.0%) were in female participants. There were 47 TB cases in Tibetan schools (394 cases/100,000 persons) and 36 cases in monasteries (235 cases/100,000 persons). The rates of newly diagnosed TB in Mundgod (486 cases/100,000 persons) and Uttarakhand (721 cases/100,000 persons) monasteries were much higher than in Bylakuppe (53 cases/100,000 persons) and Himachal Pradesh (94 cases/100,000 persons) monasteries. Two cases were identified at the reception center, and 11 cases were identified through screening of community members who came to our ACF outreach activities. No cases of TB were identified in nunneries. Because additional laboratory testing to determine the strain of *M. tuberculosis* for each of these cases was not performed, clustering of similar strains could not be assessed.

Of the 96 TB patients identified, 81 had pulmonary TB; 65 (80.2%) of those patients had positive results by sputum smear or Xpert MTB/RIF. Fifteen patients had extrapulmonary TB, and 8 patients had both pulmonary and extrapulmonary TB. A total of 5 (5.2%) patients with MDR TB (5.2%) were identified by Xpert MTB/RIF; 3 of these patients reported a history of TB. One patient with extrapulmonary TB who did not respond to first-line anti-TB treatment was later identified as having MDR TB on the basis of an Xpert MTB/RIF test of ascitic fluid that showed a positive result for rifampin resistance.

A total of 31 (32%) cases detected were positive by sputum smear, and 34 (35%) were negative by sputum smear but positive by Xpert MTB/RIF (Table 2). Twenty (19%) patients with confirmed TB had no symptoms at the time of screening (TB contacts or history of TB), and 40 patients (42%) denied having a cough.

**Table 2:** Results of microscopy for AFB and Xpert MTB/RIF test for detection of tuberculosis in 96 Tibetan refugees, India*

Results	No. (%) cases
Sputum AFB positive	31 (32.3)
Sputum AFB negative, Xpert MTB/RIF positive	34 (35.4)
Sputum AFB negative, Xpert MTB/RIF negative	16 (16.7)
Extrapulmonary TB (no sputum sample available)	15 (15.6)

*AFB, acid fast bacilli; MTB, *Mycobacterium tuberculosis*; RIF, rifampin; TB, tuberculosis.

Patients with a known close contact (within the previous 6 months) accounted for 45% (59) of all cases identified. A total of 56 (59%) patients reported contact with a person with TB at any time in the past. Nineteen (20%) patients had a previous history of TB, and only 1 person had HIV. All 96 patients were given anti-TB treatment, and 51% (49) had completed treatment by the end of the study; all other patients were still receiving treatment.

## Discussion

This study confirms that the rate of TB remains high in the Tibetan refugee population in India; the overall prevalence of undiagnosed disease was 346 cases/100,000 persons in the population screened. Our results document that estimated TB prevalence rates are high in schools (394 cases/100,000 persons) and monasteries (235 cases/100,000 persons). It was unclear why we found unexpectedly large variation between case rates for TB in monasteries in different Tibetan settlements. This finding might be caused, in part, by differences in persons seeking care from local private physicians. Fewer monks than expected were screened in Bylakuppe, where the detected case rate was low, and many monks reported that a local private physician was providing TB testing and treatment to residents. Use of private physicians is less common in Himachal Pradesh, where most Tibetan residents use the Tibetan services, including the Delek Hospital, for treatment. Further exploration into the causes of this variation would potentially be useful to improve TB control.

Another factor that might have contributed to lower than expected screening numbers in some locations was high mobility of the Tibetan population. Persons move throughout India for work, for religious pilgrimage, and to visit family. This finding had a substantial effect on population size in different locations throughout the year and was a challenge during ACF screening at the monasteries in Bylakuppe and Mundgod. Patient mobility was also a major challenge for ensuring continuity of care for receiving TB treatment. During ACF activities in Bylakuppe, many monks were on pilgrimage for the annual Kalachakra celebration.

Implementation of ACF with Xpert MTB/RIF enabled early and rapid detection of undiagnosed TB cases in Tibetan congregate living settings and resulted in diagnosis of additional cases of pulmonary TB not identified by routine sputum smear microscopy. Through ACF outreach activities conducted during this study, we identified an additional 96 cases of TB. Although it was likely that many of these patients would have eventually come to a Tibetan health center or other health facility because of signs and symptoms of TB, many persons had minimal or no symptoms, and nearly half reported no cough at the time of screening. These results suggest that ACF played a major role in identifying cases earlier in the course of disease, which might have resulted in lower subsequent transmission. The 11 case-patients identified in the community were all symptomatic at the time of evaluation and came later for treatment in the course of their disease than other patients identified in congregate settings.

One limitation of this study was that different recruitment methods were used for identifying symptomatic persons in large (>1,000 persons) and small (<1,000 persons) congregate settings, which might have had an effect on our ability to detect differences in prevalence between different congregate settings. However, because the proportion of small versus large congregate settings in settlements in Bylakuppe and Mundgod were similar, this factor probably does not affect differences in prevalence between these settlements. A second limitation was that asymptomatic persons not identified as a TB contact or with known history of TB were not evaluated in this study.

ACF was time-intensive and required major human resource capacity for screening and testing with Xpert MTB/RIF. To accommodate ACF activities, limit effects on routine medical services, and improve testing efficiency, we recruited an additional laboratory technician at each testing site. Lack of a laboratory technician has been a well-documented obstacle to implementing Xpert MTB/RIF in many settings and will be an ongoing issue for the CTA DOH if scale-up of ACF or Xpert MTB/RIF is planned. We also recruited and trained healthcare staff to provide TB education, assist with TB screening, and perform contact tracing. These workers continue to perform these activities at the health centers for which they work, demonstrating that this project also helped to build capacity of the TB program itself.

A formal cost analysis was beyond the scope of this study, but the estimated direct costs of the ACF activities was US $1,000–$1,200/case identified. However, benefits and disability-adjusted life years could not be calculated, and true cost-effectiveness is not known.

This study targeted school children, monks, and nuns who live in congregate settings and are known to be at higher risk for acquisition of TB. High rates of TB in these settings were confirmed in this study, but we also identified many TB cases in the general Tibetan community. In 2011, 42% of all TB cases registered at the Tibetan Delek Hospital were persons living in the community. During this study, 11 of 96 TB patients identified lived in the settlement community and not in a congregate living setting. Because only a small number (73) of community members participated in the screening, this finding indicates a high prevalence of TB in this subgroup and suggests that symptomatic persons took advantage of the campaign to obtain a diagnosis.

Although local staff reported increasing awareness of TB among Tibetans in India, the disease still carries a major negative stigma. For many Tibetans, particularly those with MDR TB, having TB means being isolated from daily activities (i.e., school, work, religious practices). Educational campaigns continue to be a large priority for the CTA DOH and will be essential for ongoing TB control in the Tibetan community and congregate living settings. Furthermore, efforts to provide TB screening for the general Tibetan community will be essential for TB control in this population.

We identified only 5 case-patients with MDR TB (5% of all case-patients identified); 3 of these case-patients had a history of TB. These rates are lower than expected on the basis of rates previously documented in the Tibetan refugee population, including a recent drug resistance survey among Tibetans in India, for reasons that are unclear (*14*).

In summary, the burden of TB remains high among Tibetan refugees in India. ACF and implementation of Xpert MTB/RIF has been a successful strategy for increased case detection in congregate settings in which TB diagnosis would otherwise likely have been delayed or not given. Ongoing efforts with periodic ACF by using Xpert MTB/RIF or similar rapid molecular tests, along with contact tracing and infection control measures, are warranted and will be essential for control of TB in this population.
